# Distance and Helical Phase Dependence of Synergistic Transcription Activation in *cis*-Regulatory Module

**DOI:** 10.1371/journal.pone.0031198

**Published:** 2012-01-27

**Authors:** Qilai Huang, Chenguang Gong, Jiahuang Li, Zhu Zhuo, Yuan Chen, Jin Wang, Zi-Chun Hua

**Affiliations:** 1 The State Key Laboratory of Pharmaceutical Biotechnology and Affiliated Stomatological Hospital, Nanjing University, Nanjing, People's Republic of China; 2 The State Key Laboratory of Quality Research in Chinese Medicine and Macau Institute for Applied Research in Medicine, Macau University of Science and Technology, Macau, People's Republic of China; 3 Changzhou High-Tech Research Institute of Nanjing University and Jiangsu TargetPharma Laboratories Inc., Changzhou, People's Republic of China; University of Georgia, United States of America

## Abstract

Deciphering of the spatial and stereospecific constraints on synergistic transcription activation mediated between activators bound to *cis*-regulatory elements is important for understanding gene regulation and remains largely unknown. It has been commonly believed that two activators will activate transcription most effectively when they are bound on the same face of DNA double helix and within a boundary distance from the transcription initiation complex attached to the TATA box. In this work, we studied the spatial and stereospecific constraints on activation by multiple copies of bound model activators using a series of engineered relative distances and stereospecific orientations. We observed that multiple copies of the activators GAL4-VP16 and ZEBRA bound to engineered promoters activated transcription more effectively when bound on opposite faces of the DNA double helix. This phenomenon was not affected by the spatial relationship between the proximal activator and initiation complex. To explain these results, we proposed the novel concentration field model, which posits the effective concentration of bound activators, and therefore the transcription activation potential, is affected by their stereospecific positioning. These results could be used to understand synergistic transcription activation anew and to aid the development of predictive models for the identification of *cis*-regulatory elements.

## Introduction

DNA-binding transcription factors interpret the genomic regulatory code by binding to specific sequences to activate or repress gene expression [1]. It has been thought that synergism of multiple activators in transcription initiation is an effective strategy to achieve cell diversity and dynamic response to stimuli with a limited repertoire of transcription factors [2], [3]. Increasingly, researches have shown that the understanding of the combinatorial nature of *cis*-regulatory modules is necessary for the decoding of transcription regulation networks, prediction of transcription factor binding sites, profiling of gene expression and promoter design for custom gene expression patterns [4], [5], [6], [7], [8].

The identification of *cis*-regulatory transcription elements has been a major and formidable challenge in molecular biology [9]. Development of computational methods has been attractive to overcome arduous laboratory procedures [10] and numerous tools have been developed for this task [9], [11], [12], [13], [14], [15], [16], [17]. These methods may allow the identification of *cis*-regulatory elements with impressive accuracy by utilizing constraints on motif composition/transcription factor binding affinity, orientation and relative position in the modules/regulons [18], [19], [20], [21], [22]. The probabilistic model developed by Beer and Tavazoie (2004) encoded all the constraints on a motif, exemplified by PAC (polymerase A and C box) and RRPE (ribosomal RNA processing element), including its presence, orientation, distance to the transcription start/ATG start codon, functional depth (PWM score cut-off for closeness of to “consensus”) and the presence of other motifs. However, in most predictive algorithms, the combinatorial parameters of two or more transcription factor binding sites were constrained simply by the distance between them, or by their relative distance to the core promoter [4], [18], [23].

The mechanism underlying how synergism arises has been widely explored and is ascribed to two aspects: cooperative DNA binding of two activators [24], [25] or simultaneous contact of multiple activators with the transcription initiation complex [26], [27]. Experimental studies have shown that the transcription activation function of several activators was constrained by the distance between the activator binding site(s) and the TATA box, due to distance requirements for protein-protein interactions. Transcription activation by eukaryotic activators usually decreases with increasing distance between the binding site(s) and the TATA box, including for small GAL4 derivatives [28], SP1 [29], [30], Pit-1 [31], FNR [32], and CRP [33]. Pearce et al. found that synergistic transcription by glucocorticoid receptor (GR) and AP1 was determined by the specific spacing between the GRE and AP1 sites [34].

It has been commonly believed that two DNA-bound transcription factors must occupy the same side of the DNA double helix to allow for potential interactions in regulating transcriptions [8], [35], [36], [37]. In comparison to the studies on the distance requirements of activation, few computational or experimental studies have explicitly focused on the importance of the relative stereospecific positioning, or binding face on helical DNA, of activators for synergistic transcription. Yokoyama *et al.* 2009 embedded the distance plus helical phase/stereospecificity relationships between motifs into the computational detection of known and novel *cis*-regulatory modules. Another study demonstrated that for the two prokaryotic activators FNR and CRP, transcription activation requires stereo-specific positioning of the activator and RNA polymerase on the DNA double helix [32], [33]. However, the dependence on the distance and helical phase for determining synergistic transcription activation has not ever been systematically explored in experiment and remains poorly studied or even improperly understood.

In this work, we explicitly tested the dependence of relative distance and stereospecificity of bound transcription activators on synergistic transcription. We systematically engineered binding sites for the GAL4-VP16 fusion and ZEBRA transcription activators such that their positioning around the DNA double helix varied, as suggested from reconstructions from crystal structures. These constructs were tested for activation potential and the results suggested to us that the specific positioning of activators plays a role in the recruitment of the transcription initiation complex.

## Results

### GAL4-VP16 dimers synergized for activation less effectively when bound to the same side of DNA

**Figure 1:**
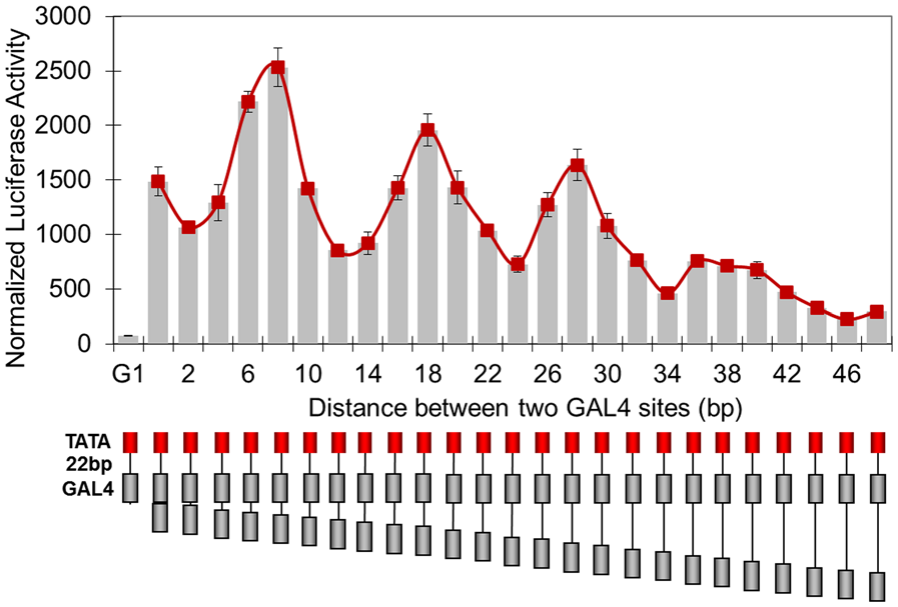
Distance dependence of transcription activation by two GAL4-VP16 dimers. GAL4 binding sites (gray box) with designed spacing from 0 to 48 bp, in steps of 2 bp, were placed 22 base pairs upstream of the TATA box of adenovirus early gene 4 (red box), followed by the coding sequence of luciferase gene. Normalized luciferase activity is plotted versus GAL4 binding site spacer length and shows local peak values at 8, 18, 28 and 36 bp. Each data point and error bar came from three parallel replicates. Each experiment was repeated twice.

GAL4-VP16 is a classical eukaryotic transcription activator due to its transcription activation potency and well-characterized DNA binding preference [28], [38], [39]. We used this transcription factor to test the transcription activation properties of various organizations of GAL4 binding sites. Our first experiment involved placing two GAL4 sites 22 base pairs upstream to the basic promoter of the adenovirus E4 gene, which initiates very weak transcription in the absence of bound upstream activators. In a series of templates, the distance between two GAL4 sites was increased from 0 to 48 bp in 2 bp steps to evaluate the effect of spacer length between GAL4 sites on transcription activation (Figure 1). The templates were transfected into 293T cells and a luciferase reporter assay was performed. We observed that luciferase activity from every template containing two GAL4 sites is obviously greater than twice of that from G1 template. It means that whatever spaced, two GAL4-VP16 dimers can always initiate transcription synergistically. In addition, overall, increasing distance between two GAL4-VP16 dimers caused the attenuation of activation. More importantly, luciferase activity varied in a sinusoidal manner as a function of increasing spacer length.

**Figure 2:**
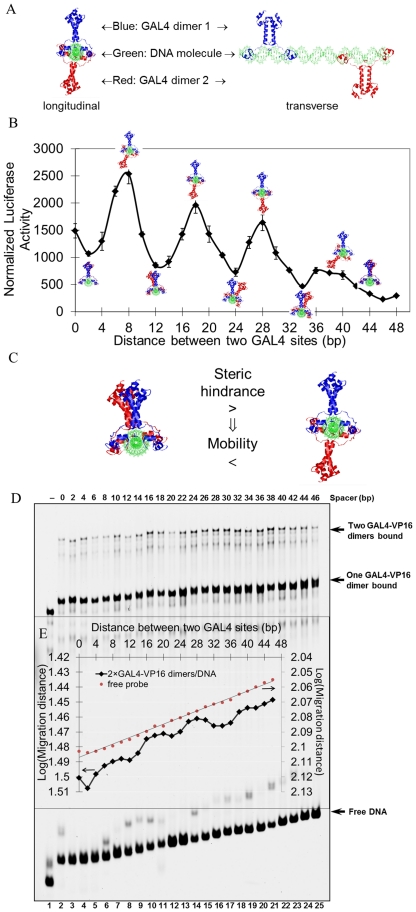
Helical phase dependence of transcription activation by two GAL4-VP16 dimers. A) Structural reconstruction of binding modes of two GAL4-VP16 dimers on the designed adenovirus promoter with two GAL4 binding sites. The GAL4-DBD dimers are shown in cartoon representation, from the experimental coordinates in PDB code 3COQ [40], bound to the promoter region. Two views are shown, longitudinal (left) and transervse (right). B) Overlay of structural reconstructions on the luciferase activity assay from Figure 1. C) Rationale for EMSA assay. Longitudinal views of structural reconstruction of two GAL4-VP16 dimers bound to promoter, left = dimers bound on the same face of DNA, right = dimers bound on opposite faces of DNA. D) EMSA. Lanes refer to experiments completed with templates increasing spacer length between GAL4 binding sites. E) Plot of logarithm(migration distance of saturated or free DNA probes in EMSA) versus GAL4 spacer length. The local minima of binding complex mobility were proven to be at separation distances of 8, 18, 28 bp and 38/40 determined according to the mobility of free probes as marker line. Note the inverted scale of log(migration distance).

Given the helical structure of double-stranded DNA, it has been suggested that increasing spacing between the two GAL4 sites will bring about periodic changes of relative phase between the two bound GAL4-VP16 dimers [28]. To evaluate whether relative phase played a role in our first experiment, we determined the spatial relationship between the two DNA bound GAL4-VP16 dimers at the various spacer lengths explored in Figure 1. HyperChem 8.0 software was employed to construct the structure of these DNA molecules, which were then aligned with the GAL4-DNA crystal structure (PDB code 3COQ) [40] (Figure 2A, more complex structures are available in Figure S1). Even though the protein-DNA complex structures with multiple GAL4 dimers have not been dissected by X-ray crystallography or NMR yet, however, we can reasonably infer that DNA fragment between the two dimers seems more likely to adopt linear but not curving shape based on the non-cooperative DNA binding property of GAL4 and ZEBRA. DNA molecule itself is intrinsic linear without external force. It is believed that some transcription factors can bend DNA when they bind, as indicated in the crystal structure of binding complex 3O9X [41], 1R8D [42], 1K6O [43], 1AKH [44] and 2PUC [45]. This bend may play a key role in the function of enhancer in initiating transcription. However, GAL4 binding does not alter the liner or near-linear conformation of DNA as indicated in the crystal structures 3COQ and 1D66 [46] containing GAL4 DNA binding domain with dimerization domain, and 1PYI [47] containing GAL4 like transcription factor PPR1. In addition, since multiple GAL4 dimers binding to naked DNA is neither cooperative nor impeditive [48], it indicates that directly physical interaction between the two GAL4 dimers seems not to exist. Similarly, activator ZEBRA also binds to multiple DNA sites without exhibiting cooperativity [49]. Without possible external force, we are likely to assume the DNA molecules are linear or near linear shape. So, it is reasonable to determine the spatial relationship of the two bound GAL4-VP16 dimers based on the linear DNA model. As shown in Figure 2B, the peaks of the sinusoidal transcription attenuation curve coincided with two GAL4-VP16 dimers bound on opposite sides of the DNA double helix, at GAL4 binding site spacer lengths of 8, 18, 28 and 36 bp in the structural reconstruction. On the contrary, troughs of the curve correlated with two GAL4-VP16 dimers bound at the same side of the DNA double helix.

We undertook EMSA experiments to validate the structural reconstruction. EMSA experiment has been largely used in the study of protein-induced DNA bending or intrinsic DNA curvature [50], [51], [52]. The greater and the closer to DNA center, the bending makes it slower for the DNA to migrate in the polyacrylamide gel. The bending angel of DNA is then determined based on the EMSA shift. Under the similar hypothesis, a protein-DNA complex of two dimers bound on opposite faces of the DNA occupied a larger volume than the protein-DNA complex in which the two activators bound on the same side of the DNA (Figure 2C), assuming a fixed orientation of the activators in the experiment. The former should migrate slower in an EMSA assay. The results indicated that along with increasing the spacer length between the two GAL4 sites from 0 to 46 bp, i.e. the increasing of the overall length of the DNA, the mobility of free DNA probe decreased gradually (Figure 2D). Significantly, the curve of the mobility of the complexes in which both GAL4 sites were fully saturated exhibited periodic fluctuation (Figure 2E). Along with the extension of the separation between two GAL4 binding sites, the DNA probe used for EMSA is getting longer progressively. It means that both the DNA length and the spatial distribution of the two bound GAL4 dimers would determine the electrophoresis mobility jointly. The effect of the DNA length has to be deprived to elucidate the spatial distribution of bound activators in the binding complex by electrophoresis mobility. So, the mobility of free probes was also dotted and lined in Figure 2E to serve as a marker for the judgment of local minima. As shown in the Figure 2E, the local minima should be the point which locally closest to the marker line. In the judgment of peaks, if three points are almost the same near to the marker line, it is reasonable to pick the middle one as the minima. For example, among the three points, 6 bp, 8 bp, 10 bp which are almost the same near to marker line, 8 bp are selected to be the minima. The periodic fluctuation showed local minima at separation distances of 8, 18, 28 bp and 38/40, indicating the protein-DNA complex had lower mobility at these distances. This result is in agreement with the structural reconstructions which showed the GAL4-VP16 dimers are on opposite faces of the DNA at these spacing values, and demonstrated that the structural reconstructions were effective in designing specific orientations of bound GAL4-VP16 activators.

Taking the luciferase-based transcription activation assay, the structural reconstructions and the mobility shift assay data together, the results suggested that transcription activation by two GAL4-VP16 dimers was most effective when they were bound on opposite faces of DNA double helix.

### The distance and helical dependence was not affected by the steric relationship between GAL4-VP16 dimers and transcription machinery

**Figure 3:**
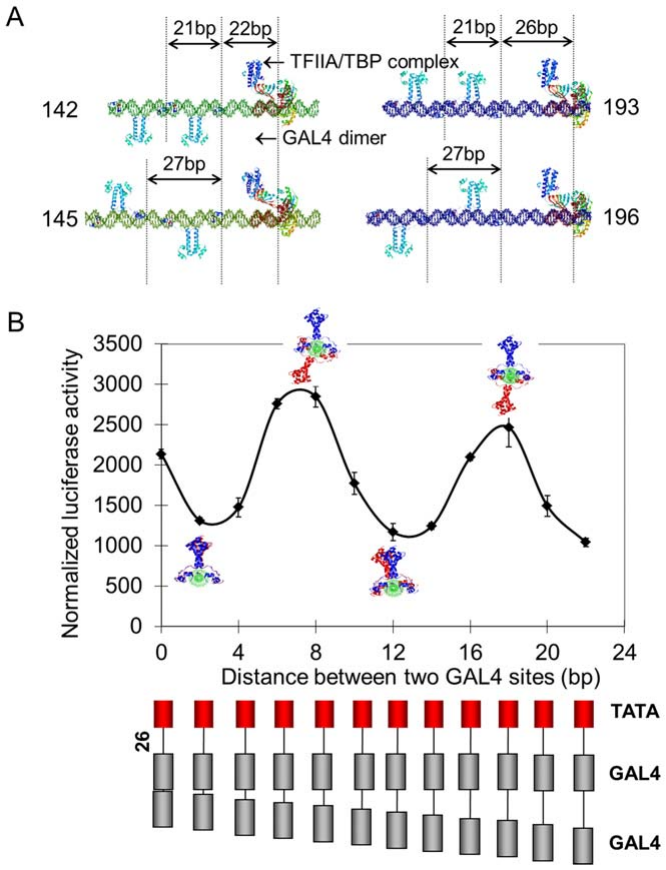
Helical phase dependence was not affected by the spatial relationship between activators and transcription complex. A) Structural reconstruction of promoter occupied by TFIIA/TBP complex and two GAL4-VP16 dimers. Four templates, 142, 145, 193 and 196, are shown. The distance between the proximal GAL4 binding site is 22 bp in templates 142 and 145, and 26 bp in templates 193 and 196. B) Luciferase activity assay using a series of transcription templates bearing two GAL4 binding sites (gray box) with increasing spacer length, with the proximal GAL4 binding site placed 26 base pairs upstream of the TATA box of adenovirus early gene 4 (red box). Each data point and error bar came from three parallel replicates. Each experiment was repeated twice.

The previous results supported a model where two activators bound to the same face of the DNA activated transcription less effectively than when bound on opposite faces. However, the importance of the spatial and stereospecific relationship between the activators and the transcription pre-initiation complex (TFIIA/TBP bound to TATA box) was unclear. To explore this, we varied the distance and orientation of the GAL4-VP16 dimers relative to the transcription complex. We constructed a series of DNA structures that bear a TATA box 22 or 26 bp downstream from the second/proximal of the two GAL4 sites, plus a varied separation distance between the two GAL4 sites. The resulting DNA molecules were then overlapped with the DNA chain in the GAL4-DNA crystal structure and then with the DNA chain in the TFIIA/TBP/TATA-box complex crystal structure (PDB 1RM1) (Figure 3A). It is notable that at separation distances of 22 and 26 bp, the TFIIA/TBP complex and the closer GAL4 dimers bound on opposite, and the same faces of the DNA double helix, respectively. Template 142 contains two GAL4 sites 21 bp spaced center-to-center, located 22 bp upstream to TATAbox. On template 142 two GAL4-VP16 dimers should bind on the same side of DNA helix and to the opposite faces to the TFIIA/TBP complex. In template 145, the two GAL4 sites were center-to-center separated by 27 bp, the two GAL4-VP16 dimers bound on opposite faces relative to each other, with the distal GAL4-VP16 dimer bound on the same side of the DNA helix as the TFIIA/TBP complex. Template 193 contains two GAL4 sites 21 bp spaced center-to-center, located 26 bp upstream to TATAbox. On template 193 two GAL4-VP16 dimers and the TFIIA/TBP complex should bind on the same side of DNA double helix. Template 196 contains two GAL4 sites 27 bp spaced center-to-center, located 26 bp upstream to TATAbox. On template 196 the proximal GAL4-VP16 dimers should bind on the same side of DNA helix to TFIIA/TBP complex, but opposite to the distal GAL4-VP16 dimers. By the luciferase activity assay (Figure 3B), we observed that transcription activation exhibited sinusoidal attenuation with increasing spacer length. Transcription activation from template 145 was higher than that from template 142, and also higher activation from template 196 was observed than from template 193 (two-fold). Therefore, an alternating binding arrangement of the two GAL4-VP16 dimers, rather an all factors bound on the same face of the DNA, results in optimal transcription activation.

### An evenly spaced distribution of multiple activators around DNA provides more effective activation

**Figure 4:**
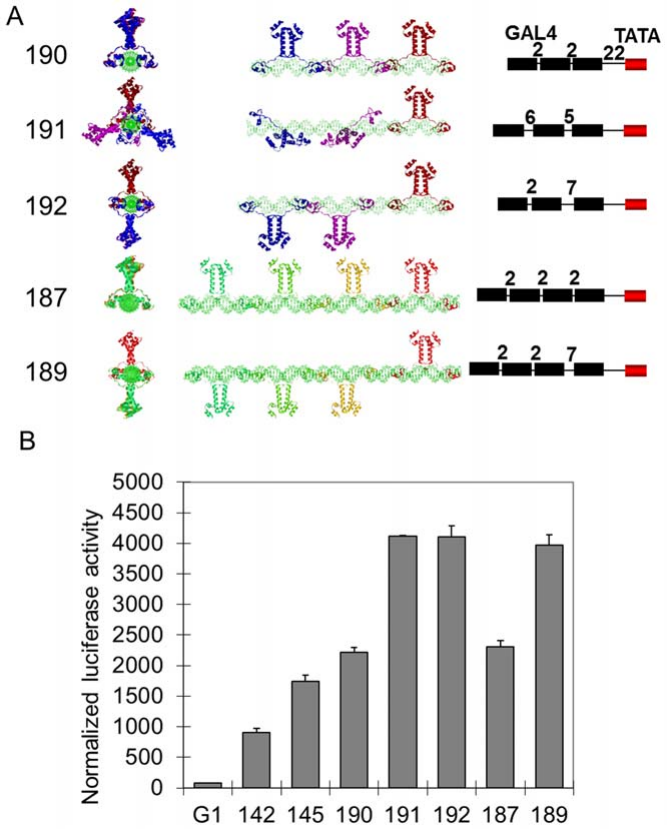
Multiple evenly distributed activators around DNA double helix function more effectively. A) Structural reconstructions of templates. All five templates, 190, 191, 192, 187 and 189, contain a proximal GAL4 binding site 22 bp upstream to the TATA box. Templates 190, 191 and 192 contain two additional GAL4 binding sites, designed to have GAL4-VP16 dimers bound on the same side of the DNA double helix (template 190), arranged regularly spaced around the DNA (template 191), or opposite from the proximal GAL4-VP16 (template 192). Templates 187 and 189 contain three additional GAL4 binding sites, with three arranged on the same face of the DNA as the transcription complex (template 187), or all four arranged opposite (template 189). B) Transcription activation assay with templates 142, 150, 190, 191, 192, 187 and 189. Each data point and error bar came from three parallel replicates. Each experiment was repeated twice.

We were interested in investigating whether the stereospecificity dependence by transcription activators extends to a greater number than two bound activators. Five transcription templates (190, 191, 192, 187 and 189) were constructed and all contained a proximal GAL4 binding site 22 bp from the TFIIA/TBP/TATA-box complex such that this GAL4-VP16 was bound on the opposite face of the DNA from the transcription complex (Figure 4A). Templates 190, 191 and 192 tested the effect of changing the relative orientation of three bound GAL4-VP16 dimers. Template 190 contained three GAL4 sites for binding of all GAL4-VP16 dimers on the same face of the DNA. Template 191 contained three GAL4 sites such that GAL4-VP16 dimers are evenly spaced around the DNA double helix (120°). Template 192 contained three GAL4 sites on which the proximal GAL4-VP16 is bound opposite to the two distal GAL4-VP16 dimers.

Template 187 contained four GAL4 sites, on which all four GAL4-VP16 dimers would reside on the same side of the DNA. Lastly, template 189 contained four GAL4 sites designed such that the proximal GAL4-VP16 binds opposite to the transcription complex, and the other three GAL4-VP16 dimers bound on the same side of the DNA relative to the transcription complex.

The luciferase reporter assay using these templates demonstrated that transcription activation from templates 191 and 192 were both equal and much greater than that from template 190 (Figure 4B). This illustrated that an identical relative arrangement of activators, as in template 190, is not effective for transcription activation. Interestingly, a regular spacing of activators around the DNA double helix, as in template 191, did not increase activation further as compared to alternating the proximal and the next activator, as in template 192. This is reflected in the fact that activation from template 189 is greater than from 187, where the increased activation was observed when the first two dimers were arranged oppositely.

**Figure 5:**
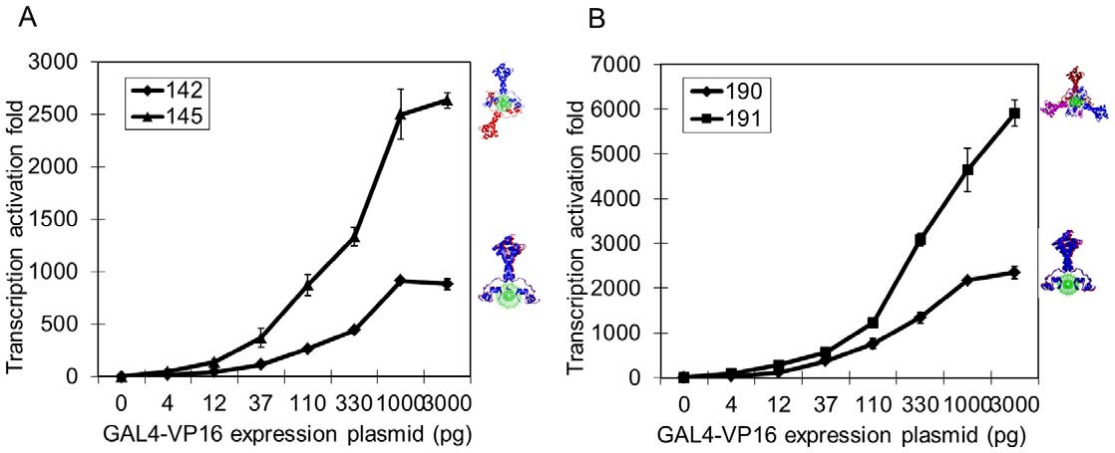
Evaluation of concentration dependence. Evenly distributed activators around the DNA double helix activate transcription more effectively at all activator concentrations. Transcription activation assays were completed with templates 142, 145 (A), 190 and 191 (B), with increasing concentration of GAL4-VP16 expression plasmid in the luciferase assay. Each data point and error bar came from three parallel replicates. Each experiment was repeated twice.

To rule out the involvement of activator concentration in our results, the concentration of GAL4-VP16 was varied by increasing the amount of expression plasmid included in the cell transfection. Transcription activation increased with increasing levels of GAL4-VP16 expression plasmid for all of the transcription templates (142, 145, 190 or 191, Figure 5). Template 145 stimulated transcription more than template 142 at all levels of GAL4-VP16 expression plasmid (Figure 5A). Similarly, template 191 activated transcription to a higher level than template 190 at all levels of expression plasmid (Figure 5B). These results show that the level of expression plasmid is correlated with the level of transcription activation, however, at no level of expression plasmid was the activation level from templates designed for binding of GAL4-VP16 dimers all on the same side of DNA higher than the activation from templates designed for GAL4-VP16 binding in opposite/regularly spaced arrangement around the DNA. This conclusion is consistent with our earlier observations and rules out a concentration dependence.

### ZEBRA also more effectively activated transcription when arranged around the DNA double helix

**Figure 6:**
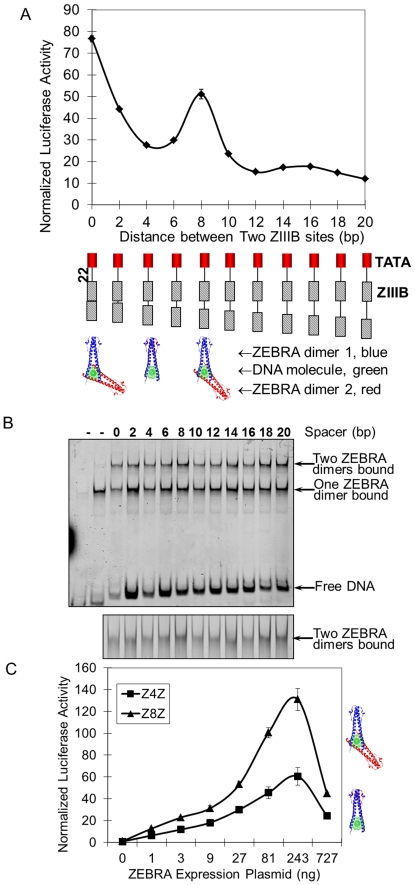
Distance and phase dependence of synergistic transcription activation by two ZEBRA transcription activators. Two ZEBRA binding sites (ZIIIB, oblique-line box) with increasing spacers length (in steps of 2 bp) were placed 22 bp upstream to the TATA box (red box). A) Transcription activation luciferase assay using templates designed with increasing distance between ZIIIB sites. B) EMSA assay. Lanes show experiments completed with templates designed with increasing distance between ZIIIB sites. Below the main gel is zoom in of region of the gel showing two ZEBRA molecules bound. C) Transcription activation assays were completed with templates Z4Z (ZIIIBs were 4 bp spaced) and Z8Z (8 bp spaced), with increasing concentration of ZEBRA expression plasmid in the luciferase assay. Each data point and error bar came from three parallel replicates. Each experiment was repeated twice.

The above results could be specific for the VP16 activation domain. To study this possibility, we repeated the analysis with ZEBRA, belonging to a different class of transcription activator. The transcription activation domain of ZEBRA is rich in glycine, proline and glutamine residues [53], whereas VP16 belongs to the class of factors rich in acidic residues [54]. We constructed a series of templates with increasing distance between two ZIIIB sites. Using the luciferase assay, we found that overall, transcription activation decreased with increasing of length of spacing between the ZIIIB sites, except for a peak of activation at spacing of 8 bp (Figure 6A). To corroborate these results, we repeated the analysis using an EMSA assay (Figure 6B). The experiment showed that the highest level of mobility retardation was observed of a complex of two ZEBRA dimers separated by 8 bp. This suggested that the protein-DNA complex at this separation occupied the largest volume. We reconstructed this protein-DNA complex using the PDB ID 2C9L [55] at this separation distance. The structural reconstruction showed at this spacing, two ZEBRA dimers bound on opposite faces of the DNA double helix, consistent with the EMSA results (Figure 6A). Further investment of activator concentration dependence by increasing ZEBRA expression plasmid in cell transfection, indicated that at all activator concentrations, activation level from template (Z8Z) designed for ZEBRA binding on opposite faces of DNA helix were higher than activation from template Z4Z designed for ZEBRA binding on the same side of DNA (Figure 6C). These observations are in agreement with our results for the GAL4-VP16 system and suggest a dependence on stereospecific positioning around DNA could be a general property of transcription activators, with greater activation by bound activators arranged on opposite faces of DNA.

## Discussion

It has long been known that the structural characteristics of DNA-binding motifs place requirements on the spacing and nature of recognition sequences [30], [56]. In contrast, it has been unclear whether synergistic transcription activation requires a specific arrangement of the activators around the DNA double helix and that was the focus of this study.

We demonstrated that transcription activation magnitude varied in a sinusoidal manner with increasing spacing between engineered GAL4 binding sites. We showed by computational molecular structure reconstruction and by EMSA that the peaks in this pattern occurred when two GAL4-VP16 dimers bound on opposite faces of the DNA double helix. We also observed this phenomenon with multiple GAL4 binding sites, where a regular binding distribution of GAL4-VP16 around the DNA best activated transcription. Furthermore, we showed that the sinusoidal fluctuation of synergistic transcription activation by two GAL4-VP16 dimers was not affected by their relative helical positioning to the TFIIA/TBP transcription complex bound on TATA box. Lastly, these results were not specific for GAL4-VP16, as transcription activated by the ZEBRA transcription factor from *cis*-regulatory modules composed of two ZIIIB binding sites was maximal with ZEBRA dimers bound on opposite faces of the DNA double helix.

Our findings are supported by studies of the NFY transcription factor [8]. In *cis*-regulatory modules containing two NFY binding sites from mouse and human promoters, the center-to-center distance between these two NFY binding sites were statistically proven to prefer approximately 15, 25, 35 and 45 bp. Similarly, for the motif pairs composed of one NFY binding site and one SP1 binding site, the center-to-center distances of the two motifs were statistically proven to prefer about 15 and 25 bp. These separation distances corresponded to binding of activators on opposite faces of the DNA double helix, which we observed for our test activators. Since the interactions between two NFY proteins, or between one NFY factor and one SP1 factor, have been well studied [57], [58], it is notable that these transcriptional activators tend to bind opposite on the DNA double helix. It suggests that, our observation is general enough for all transcription activation.

In their studies, Yokohama *et al* (2009) developed the “motif relational function” (MRF) to detect spatial biases between motif-pairs using regression analysis in human and mouse promoter sequences and found that motif-pairs often co-occur preferentially at multiple separation distances corresponding to half-turn of the DNA double helix. However, those results were misinterpreted under the assumption that transcription factor interactions only occur when positioned in the same orientation around histone complex or the DNA double helix [35], [37]. They ignored and failed to discover the key character as we found in this present work. All these studies suggest that, as a strategy for organism to activate transcription most elaborately during revolution, the recognition sequences may instead be positioned at distances that allow for binding of the activators on opposite faces of the DNA.

### Current models of synergistic transcription activation

Various models have been employed to explain the distance and stereospecificity constraints on synergistic transcription activation. According to the simultaneous contact model, multiple activators bind to recognition sequences and contact simultaneously with transcription initiation complex component(s) to recruit them to assemble on the core promoter [8], [36]. Activators will drive synergistic transcription only when they are positioned on the same side of the DNA double helix. This is obviously contradictory to and cannot be used to explain what we observed in our works. An additional model is the DNA looping-out model [36]. Given that the activators and the transcription complex are tethered to DNA, this model suggests that the length of the intervening DNA sequence between the activator and the transcription complex is a factor in determining the flexibility of this sequence and the probability of interaction between the two protein complexes [59], [60]. The highest probability of interaction between two DNA tethered proteins via an intervening is reported to occur at a separation length of 500 bp, at which distance the intervening DNA between the transcription complex and the nearest bound activator can loop out and avoid steric clashes with the bound factors [60]. In our analysis, we placed GAL4/ZEBRA sites only 22 bp upstream of the TATA box. Therefore, looping out of the DNA between the TATA box and proximal activator binding site is minimal. The stereospecificity dependence we observed should not be affected by the presence of the intervening DNA and therefore the DNA looping out model is not sufficient to explain our observations.

### A novel model of synergistic transcription activation: the concentration field model

**Figure 7:**
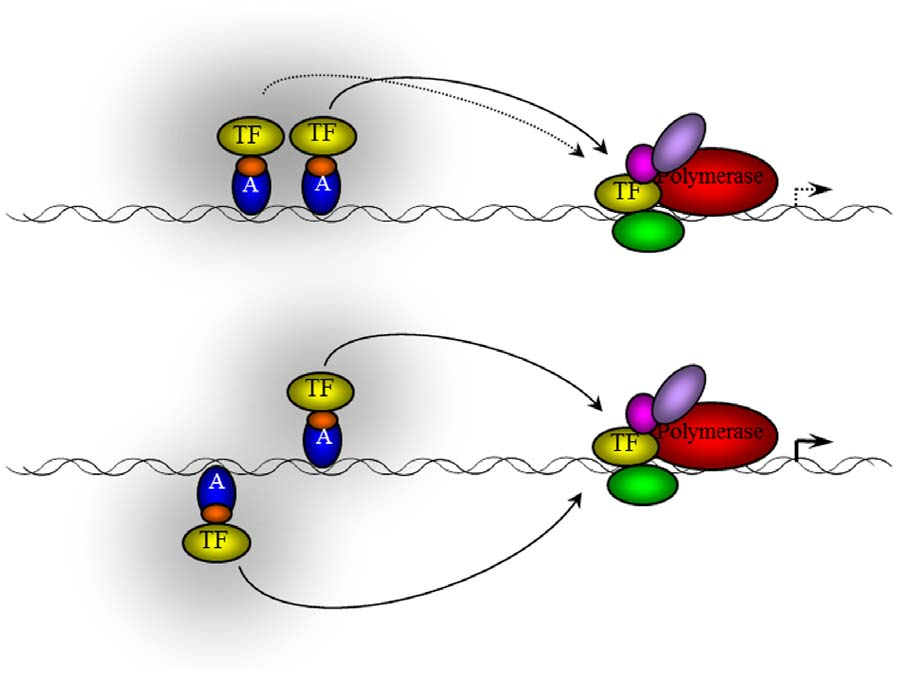
Concentration field model of transcription activation. Transcription activator binds on the promoter and recruit transcription machinery components (TF) to the TATA box to form the transcription initiation complex. If multiple activators are bound on the same side of DNA, the proximal activator provides steric hindrance to the protein-protein interactions mediated by the distal activator, therefore decreasing the total recruitment of TFs to the TATA box. Conversely, an arrangement of activators on opposite faces of the DNA, activators are free to recruit TFs.

We describe a novel model, the concentration field model, which considers the binding of transcription activators to the DNA double helix as a kinetic equilibrium of binding and dissociation events. The balance between the dynamic binding and dissociation events of activators to DNA determines the effective concentration of activator at the binding site location and therefore their activation potential (Figure 7). Transcription synergy arises from the cooperative increase of transcription initiation complex components around the TATA box by the multiple transcription activators. The model suggests that multiple activators function less efficiently for transcription activation when they are bound on the same side of the DNA double helix, since the frequency of activator binding/dissociation events at the binding site would be greater for dissociation events due to steric clashes. Similarly, the model suggests higher synergism of multiple transcriptional activators originates from the lack of steric clashes when activators are bound on opposite/regularly spaced positions around the DNA double helix.

Since many activators have been reported to interact with some component of the transcription machinery (reviewed in [61]), and that binding of the transcription complex itself can be described by binding and dissociation events, the effective concentration of the transcription machinery could have a similar stereospecificity dependence as that of the activator. Extending this model to the transcriptional complex bound at the TATA box, binding of activators in a productive series of oppositely/regularly-spaced positions around the DNA double helix could favour interactions between the activators and the transcription complex. Therefore, we suggest this model for best explaining the synergism of multiple transcriptional activators and the importance of their specific spatial positioning as observed in this work.

We are aware that this model assumes a simplistic structure of transcription factor. Many transcription factors are composed of multiple domains, with each domain exhibiting either activation or DNA-binding functionalities. These domains are often separated by flexible linkers, such as in the KRAB-zinc finger or BTB-zinc finger proteins (reviewed in [62]), and rigid body movements of the activation domain of these proteins with respect to their tethered DNA-binding domains may allow these factors to overcome any stereospecificity effects we observed. However, the GAL4-VP16 fusion protein involved in this study did have a small linker region between the activation and DNA binding domain, suggesting an increased length of the linker might be necessary to overcome our observed stereospecificity requirements. The concentration field model could best apply to transcription factors whose DNA binding and activation domains are not separated by a significant linker, as with leucine zipper activators, or factors in which these two functionalities co-exist in the same structured domain.

## Materials and Methods

### Construction of transcription templates

The transcription template plasmids pE4T, pG1E4T and pZ1E4T, bearing none, one GAL4 binding site or one ZEBRA binding site ZIIIB upstream to the −38 - +280 region of the adenovirus E4 gene promoter, were kindly provided by Prof. Michael Carey (UCLA, USA) [63]. DNA sequences containing transcription activator binding site(s) and downstream −38 – +38 region of E4 gene promoter were amplified from these three plasmids with primers E4Tup (5′-GACGGCTAGCACATACGATTTAGGTGACAC-3′) and E4Tdown (5′-GAGAAGATCTCACCACTCGACACGGCACC-3′), digested with NheI and BglII and inserted into pGL3 basic vector to construct the *in vivo* transcription templates pE4TGL3, pG1GL3 and pZ1GL3 respectively. GAL4 or ZEBRA binding sites on other *in vivo* transcription templates were generated using an iterative process from pE4TGL3, as follows and elaborated in Table S1. Template 126 was derived from pE4TGL3 by introducing one EcoRV site next to the single GAL4 binding site by site-directed mutagenesis. Transcription templates 142 to 145 were constructed by inserting annealed double-stranded DNA containing GAL4 binding sites with designed spacings between PstI and the engineered EcoRV site of template 126. Templates 146 to 151 was constructed by insertion of double-stranded DNA containing GAL4 binding sites with designed spacing between PstI and SmaI cleaved template 145. Transcription templates 156–173 and 187–203 were similarly constructed. Templates 174–186 were constructed by re-joining the 2000 bp Sal I/EcoR V double restriction fragments from each of the plasmids including 146–151 and 172–179 with the 2800 bp Sal I/SmaI double restriction fragments of plasmid 172. Accession numbers in GenBank of these new DNA sequencing data were included in Table S1.

### Construction of effector plasmids

Full length ZEBRA coding sequence was amplified from prokaryotic expression vector pET11d-ZEBRA (gift from Prof. Michael Carey of University of California, Los Angeles) using primers ZEBRAup (5′-ATCCGATATCCATGGACCCAAACTCGAC-3′) and ZEBRAdown (5′-CCCGCTCGAGTTAGAAATTTAAGAGATCC-3′). The PCR product was digested with EcoRV and XhoI and inserted into the eukaryotic expression vector pCI-HA to construct pCI-HA-ZEBRA. GAL4-VP16 coding sequence was amplified from the prokaryotic expression vector pGEX2TK-GAL4-VP16 (gift from Prof. Michael Carey of UCLA) using primers GAL4up (5′-CCCGATATCTATGAAGCTACTGTCT-3′) and VP16down (5′-CTGCCTCGAGTTACCCACCGTACTCGTCAAT-3′). The PCR product was digested with EcoRV and inserted into SmaI-linearized eukaryotic expression vector pRK5-FLAG to obtain pRK5-FLAG-GAL4-VP16. The insertion direction of GAL4-VP16 coding sequence was confirmed through XhoI cleavage. Both these two expression constructs were proven to be correct by DNA sequencing (Invitrogen, China).

### Expression and purification of GAL4-VP16

GAL4-VP16 containing residues 1–147 of Gal4 attached by an amino acid linker (PEFPGIW) to residues 413–490 of VP16 [39] were expressed as an N-terminal GST fusion protein under the control of the tac promoter by plasmids pGEX-2TK (gift from Micheal Carey, UCLA) in *Escherichia coli*. Cultures were grown at 37°C to an OD_600_ of 0.6. The expression of the fusion proteins was induced with 1 mM isopropylthiogalactoside for 3 h. Cells from 1 liter of culture were harvested, washed and resuspended in 20 ml phosphate buffered saline (PBS, containing 0.5 mM phenylmethylsulfonyl fluoride, PMSF), and lysed by sonication in an ice bath. All subsequent operations were performed at 0 to 4°C. A crude extract was derived by centrifugation of the lysate for 20 min at 12000 g and then applied to 100 µl of glutathione sepharose pre-equilibrated with PBS. After enough washing, the GST-GAL4-VP16 fusion retained on the resin was digested with 10 units of thrombin protease (Amersham Pharmacia Biotech, 27-0846-01) overnight at 4°C. The eluant was collected by centrifugation and subjected to SDS-PAGE. The protein was approximately 90% pure as judged by Coomassie blue staining of sodium dodecyl sulfate (SDS)-polyacrylamide gels.

### Expression and purification of ZEBRA

Full length ZEBRA (BamH I Z fragment, Epstein-Barr Replication Activator) [64] was expressed in *E. coli* BL21(DE3) cells under the control of the T7 promoter in plasmids pET11d (gift from Micheal Carey, UCLA) and purified as described previously [49]. The protein was approximately 90% pure as judged by Coomassie blue staining of SDS-polyacrylamide gels.

### Electrophoretic Mobility Shift Assay

DNA sequences containing two GAL4 binding sites of increasing spacing from 0 to 46 bp in 2 bp steps were amplified through PCR reaction using two primers, the fluorescently labeled 5-ROX-SP6 (ROX-5′-GATTTAGGTGACACTATAGAATAC-3′, HPLC pure, Invitrogen) and E4TATAR (5′-GCGAGTATATATAGGACTGGG-3′, Invitrogen). The PCR products were then separated on 1.5% agarose gel and recovered using 3S Spin DNA Agarose Gel Purification Kit (Biocolor BioScience & Technology Company, BBST) and kept in Tris-EDTA (TE) buffer. The 10 µl binding reaction contains 5.0 µl buffer D (20 mM HEPES-KOH (pH 7.9), 20% (v/v) glycerol, 0.2 mM EDTA, 0.1 M KCl, 0.5 mM PMSF, 1.0 mM DTT), 0.6 µl of 1 µg/µl poly(dI:dC)·poly(dI:dC) (Sigma, P4929-25U), 0.25 µl of 8 µg/µl BSA, 0.1 µl of 0.1 M DTT, 0.75 µl of 0.1 M MgCl_2_, 1.0 µl of ROX labeled DNA fragment, and 1.0 µl of ZEBRA or GAL4-VP16 protein. After incubation at 25°C for 1 h, samples were loaded onto a pre-run 20 cm long 4.5% native polyacrylamide gel. Gel was run in 0.5×TBE containing 1% glycerol at 100 V for 1 hour. After electrophoresis, images were acquired with a Typhoon 9410 imager system (Amersham Biosciences). 5-ROX fluorescence was excited with a green laser (wavelength 532 nm) and detected with 610-nm band pass filter. The EMSA data were analyzed using ImageQuant TL software.

### Cell culture and transfection

293T cells originated from ATCC were maintained in Dulbecco's modified Eagle's medium (Invitrogen, USA) supplemented with 10% (v/v) newborn bovine serum (HyClone, USA), 100 U/ml penicillin and 100 µg/ml streptomycin [65]. Plasmids were introduced into cells by M-PEI mediated transfection as described previously [66]. Briefly, cells were placed in a 24-well plate at a density of 5×10^5^ cells/ml. When grown to 50% confluence, cells were transfected with 1.0 µg M-PEI-complexed plasmids in serum-free DMEM medium. The ratio of plasmid to PEI polymer was 1∶1.5. Five h later, cells were supplied with 700 µl fresh medium containing serum but without antibiotics, and were then continuously cultured for another 31 h. For normalization of transfection efficiencies, 100 ng Renilla luciferase expression plasmid pRL-CMV was included in each transfection experiment.

### Reporter Gene Assays

The transfected cells were harvested and washed with cold PBS for 36 h post-transfection and lysed in 100 µl 1×passive lysis buffer (Promega, E194A). Insoluble debris was removed by centrifugation at 12000 g at 4 °C for 5 min. The enzyme activities of firefly luciferase and Renilla luciferase were acquired in turn according to the instruction of Dual-Luciferase® Reporter Assay System E1910 (Promega, USA). 2 µl of each lysate was mixed with 10 µl LARII to read the firefly luciferase activity and then 10 µl Stop & Glo® Reagent was added to read the Renilla luciferase activity in a 20/20^n^ luminometer (Turner Biosystems, USA). The Firefly luciferase activity was divided by Renilla luciferase activity to normalize the transcription level.

### Molecular Structure Construction and Superimposition

The structure of DNA molecules were constructed employing HyperChem 8.0 software (Hypercube Inc., USA) and optimized with Molecular Mechanics Force calculation using Geometry optimization arithmetic, for which HyperMM+ force field was selected for optimization. The sequences of each DNA molecule submitted to molecular structure construction were listed in Table S2; these constructed DNA molecules vary by the number of and spacing of transcription factor binding site(s). We employed the superimposition function of Discovery Studio 2.1 (Accelrys, USA) to construct molecular structures of protein-DNA complexes containing the transcription factors, GAL4-VP16, ZEBRA or TFIID and the DNA molecule previously constructed using HyperChem 8.0. The DNA binding site sequence on the constructed DNA molecule was overlapped to the equal binding site sequence on the DNA molecule in published crystal structures. For the overlapping of GAL4 binding site sequences, the first chain (sense chain) of the optimized constructed DNA molecule was superimposed with the D chain of the nucleotide sequence of GAL4-DNA crystal structure (Protein Databank (PDB) ID 3COQ) [40]. For the overlapping of ZEBRA binding site sequences, the first chain (sense chain) of the optimized constructed DNA molecule was superimposed with the A chain of nucleotide sequence of ZEBRA-DNA crystal structure (PDB ID 2C9L) [55]. For the overlapping of TBP binding site i.e. TATA box, the first chain (sense chain) of the optimized constructed DNA molecule was superimposed with the D chain of crystal structure of Yeast TFIIA/TBP/TATA-box DNA Complex (PDB ID 1RM1). Molecular structure figures were produced using Rasmol [67].

## Supporting Information

Figure S1 **Structural reconstruction of binding modes of two GAL4-VP16 dimers on the designed adenovirus promoter with two GAL4 binding sites.** The GAL4-DBD dimers are shown in cartoon representation, from the experimental coordinates in PDB code 3COQ [40], bound to the promoter region.
(TIF)

Table S1 **Construction strategies of transcription templates and their GenBank accession numbers.**
(DOC)

Table S2 **Information of DNA molecules used for computational structure construction.**
(DOC)
