# Supplementary figures and images for: Distance and Helical Phase Dependence of Synergistic Transcription Activation in cis-Regulatory Module
Source: PLoS One. 2012 Jan 27;7(1):e31198. doi: 10.1371/journal.pone.0031198 (PMC3267773; doi:10.1371/journal.pone.0031198)

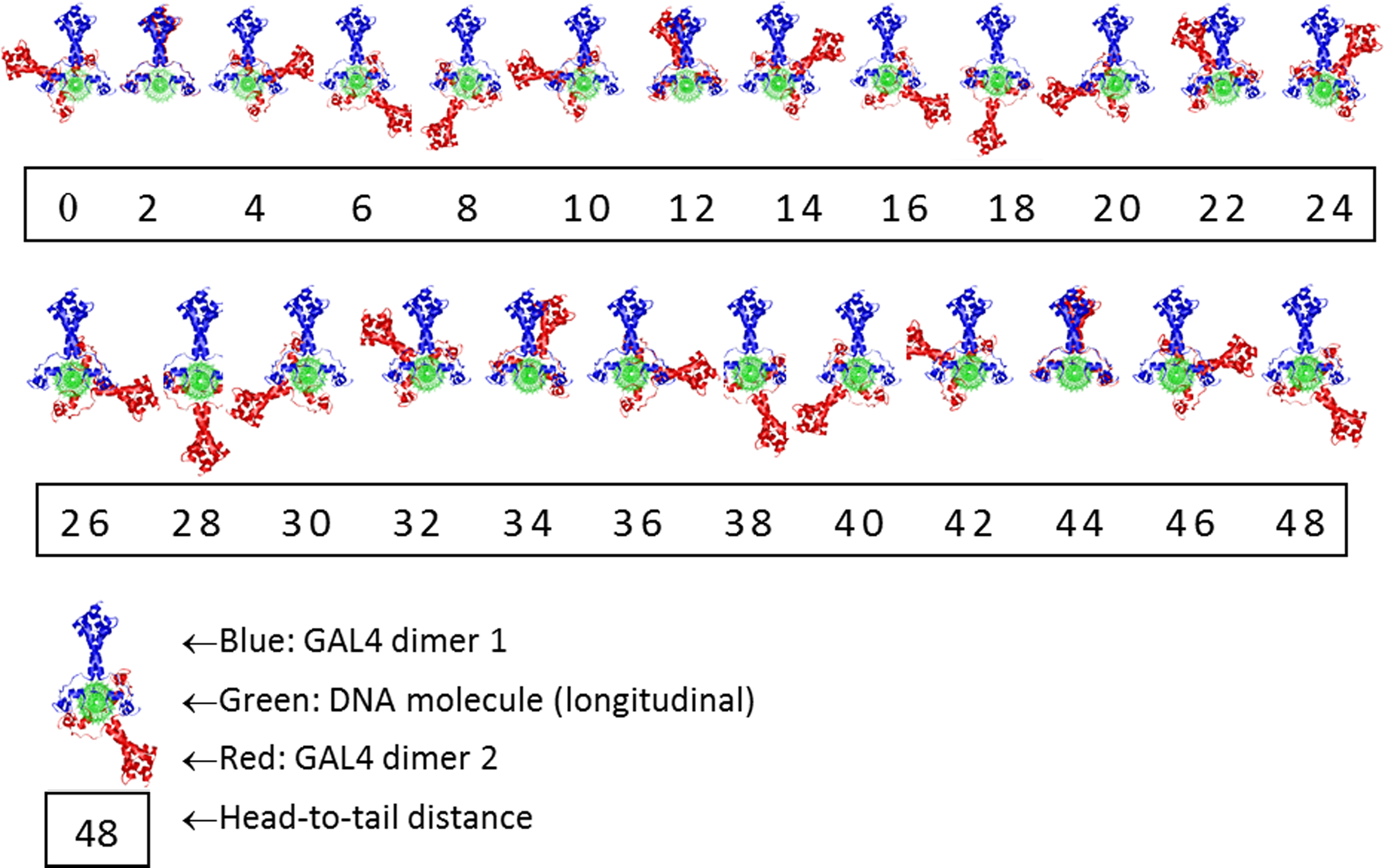

Supplement: Figure S1 — Structural reconstruction of binding modes of two GAL4-VP16 dimers on the designed adenovirus promoter with two GAL4 binding sites. The GAL4-DBD dimers are shown in cartoon representation, from the experimental coordinates in PDB code 3COQ [40], bound to the promoter region. (TIF) [file pone.0031198.s001.tif]
