# Supplementary material for: Distance and Helical Phase Dependence of Synergistic Transcription Activation in cis-Regulatory Module
Source: PLoS One. 2012 Jan 27;7(1):e31198. doi: 10.1371/journal.pone.0031198 (PMC3267773; doi:10.1371/journal.pone.0031198)
Supplement: Table S1 — Construction strategies of transcription templates and their GenBank accession numbers. (DOC) [file pone.0031198.s002.doc]

Table S1

| Template No. | Accession No. | Construction strategies |
| --- | --- | --- |
| 126 | JN244031 | Annealed and inserted between Pst I/BamH I of pE4TGL3  126F 5’-**GCTCGGAGGACAGTACTCCG**GATATCCTAGAG  126R 5’-GATCCTCTAGGATATCCGGAGTACTGTCCTCCGAGCTGCA |
| G2 | JN244032 | Annealed and inserted between Pst I/EcoR V of p126GL3  G2F **GCTCGGAGGACAGTACTCCGCTCGGAGGACAGTACTCCG**G  G2R CCGGAGTACTGTCCTCCGAGCGGAGTACTGTCCTCCGAGCTGCA |
| 142 | JN244033 | Annealed and inserted between Pst I/EcoR V of p126GL3  142F **GCTCGGAGGACAGTACTCCG**GG**CTCGGAGGACAGTACTCCG**G  142R CCGGAGTACTGTCCTCCGAGCCCGGAGTACTGTCCTCCGAGCTGCA |
| 143 | JN244034 | Annealed and inserted between Pst I/EcoR V of p126GL3  143F **GCTCGGAGGACAGTACTCCG**CCGG**CTCGGAGGACAGTACTCCG**G  143R CCGGAGTACTGTCCTCCGAGCCGGCGGAGTACTGTCCTCCGAGCTGCA |
| 144 | JN244035 | Annealed and inserted between Pst I/EcoR V of p126GL3  144F **GCTCGGAGGACAGTACTCCG**CCCGGG**CTCGGAGGACAGTACTCCG**G  144R CCGGAGTACTGTCCTCCGAGCCCGGGCGGAGTACTGTCCTCCGAGCTGCA |
| 145 | JN244036 | Annealed and inserted between Pst I/EcoR V of p126GL3  145F **GCTCGGAGGACAGTACTCCG**GCCCGGGC**CTCGGAGGACAGTACTCCG**G  145R CCGGAGTACTGTCCTCCGAGGCCCGGGCCGGAGTACTGTCCTCCGAGCTGCA |
| 146 | JN244037 | Annealed and inserted between Pst I/Sma I of p145GL3  123F **GCTCGGAGGACAGTACTCCG**GATATC  146R GATATCCGGAGTACTGTCCTCCGAGCTGCA |
| 147 | JN244038 | Annealed and inserted between Pst I/Sma I of p145GL3  124F **GCTCGGAGGACAGTACTCCG**GATATCAG  147R CTGATATCCGGAGTACTGTCCTCCGAGCTGCA |
| 148 | JN244039 | Annealed and inserted between Pst I/Sma I of p145GL3  125F **GCTCGGAGGACAGTACTCCG**GATATCAGAG  148R CTCTGATATCCGGAGTACTGTCCTCCGAGCTGCA |
| 149 | JN244040 | Annealed and inserted between Pst I/Sma I of p145GL3  126F **GCTCGGAGGACAGTACTCCG**GATATCCTAGAG  149R CTCTAGGATATCCGGAGTACTGTCCTCCGAGCTGCA |
| 150 | JN244041 | Annealed and inserted between Pst I/Sma I of p145GL3  127F **GCTCGGAGGACAGTACTCCG**GATATCCTCTAGAG  150R CTCTAGAGGATATCCGGAGTACTGTCCTCCGAGCTGCA |
| 151 | JN244042 | Annealed and inserted between Pst I/Sma I of p145GL3  128F **GCTCGGAGGACAGTACTCCG**GATATCTCCTCTAGAG  151R CTCTAGAGGAGATATCCGGAGTACTGTCCTCCGAGCTGCA |
| 162 | JN244074 | Annealed and inserted between Pst I/EcoR V of p126GL3  162F **GGCCACATTAGCAAT**GCCGG**GCCACATTAGCAAT**GCCG  162R C GGCATTGCTAATGTGGCCCGGCATTGCTAATGTGGCCTGCA |
| 163 | JN244075 | Annealed and inserted between Pst I/EcoR V of p126GL3  163F **GGCCACATTAGCAAT**GCCCCGG**GCCACATTAGCAAT**GCCG  163R CGGCATTGCTAATGTGGCCCGGGGCATTGCTAATGTGGCCTGCA |
| 164 | JN244076 | Annealed and inserted between Pst I/EcoR V of p126GL3  164F **GGCCACATTAGCAAT**GCCCCCGGG**GCCACATTAGCAAT**GCCG  164R CGGCATTGCTAATGTGGCCCCGGGGGCATTGCTAATGTGGCCTGCA |
| 165 | JN244077 | Annealed and inserted between Pst I/EcoR V of p126GL3  165F **GGCCACATTAGCAAT**GCCGCCCGGGC**GCCACATTAGCAAT**GCCG  165R CGGCATTGCTAATGTGGCGCCCGGGCGGCATTGCTAATGTGGCCTGCA |
| 166 | JN244078 | Annealed and inserted between Pst I/Sma I of p165GL3  166F **GGCCACATTAGCAAT**GCCGATATC  166R GATATCGGCATTGCTAATGTGGCCTGCA |
| 167 | JN244079 | Annealed and inserted between Pst I/Sma I of p165GL3  167F **GGCCACATTAGCAAT**GCCGATATCAG  167R CTGATATCGGCATTGCTAATGTGGCCTGCA |
| 168 | JN244080 | Annealed and inserted between Pst I/Sma I of p165GL3  168F **GGCCACATTAGCAAT**GCCGATATCAGAG  168R CTCTGATATCGGCATTGCTAATGTGGCCTGCA |
| 169 | JN244081 | Annealed and inserted between Pst I/Sma I of p165GL3  169F **GGCCACATTAGCAAT**GCCGATATCCTAGAG  169R CTCTAGGATATCGGCATTGCTAATGTGGCCTGCA |
| 170 | JN244082 | Annealed and inserted between Pst I/Sma I of p165GL3  170F **GGCCACATTAGCAAT**GCCGATATCCTCTAGAG  170R CTCTAGAGGATATCGGCATTGCTAATGTGGCCTGCA |
| 171 | JN244083 | Annealed and inserted between Pst I/Sma I of p165GL3  171F **GGCCACATTAGCAAT**GCCGATATCTCCTCTAGAG  171R CTCTAGAGGAGATATCGGCATTGCTAATGTGGCCTGCA |
| 173 | JN244043 | Annealed and inserted between Pst I/Sma I of p145GL3  173F **GCTCGGAGGACAGTACTCCG**GATATCTACTCTAGACCC  173R GGGTCTAGAGTAGATATCCGGAGTACTGTCCTCCGAGCTGCA |
| 174 | JN244044 | Sal I/ SmaI double digestion of p172GL3 to produce ~2000bp and ~2800bp fragments.  Sal I/EcoR V double digestion of p146GL3 to produce ~2000bp and ~2800bp fragments.  Join the 146/SalI/ EcoR V /2000 with 172/SalI/SmaI/2800 to produce p174GL3 (G24).  **GCTCGGAGGACAGTACTCCG**GATATCTCTCTAGACCCATCGGGC**CTCGGAGGACAGTACTCCG**GATCCTA |
| 175 | JN244045 | Sal I/ SmaI double digestion of p172GL3 to produce 2000bp and 2800bp fragments.  Sal I/EcoR V double digestion of p147GL3 to produce 2000bp and 2800bp fragments.  Join the 147/SalI/ EcoR V /2000 with 172/SalI/SmaI/2800 to produce p175GL3 (G26G).  **GCTCGGAGGACAGTACTCCG**GATATCTCTCTAGACCCATCAGGGGC**CTCGGAGGACAGTACTCCG**GATCCT |
| 176 | JN244046 | Sal I/ SmaI double digestion of p172GL3 to produce 2000bp and 2800bp fragments.  Sal I/EcoR V double digestion of p148GL3 to produce 2000bp and 2800bp fragments.  Join the 148/SalI/ EcoR V /2000 with 172/SalI/SmaI/2800 to produce p176GL3 (G28G).  **GCTCGGAGGACAGTACTCCG**GATATCTCTCTAGACCCATCAGAGGGGC**CTCGGAGGACAGTACTCCG**GATC |
| 177 | JN244047 | Sal I/ SmaI double digestion of p172GL3 to produce 2000bp and 2800bp fragments.  Sal I/EcoR V double digestion of p149GL3 to produce 2000bp and 2800bp fragments.  Join the 149/SalI/ EcoR V /2000 with 172/SalI/SmaI/2800 to produce p177GL3 (G30G).  **GCTCGGAGGACAGTACTCCG**GATATCTCTCTAGACCCATCCTAGAGGGGC**CTCGGAGGACAGTACTCCG**GATC |
| 178 | JN244048 | Sal I/ SmaI double digestion of p172GL3 to produce 2000bp and 2800bp fragments.  Sal I/EcoR V double digestion of p150GL3 to produce 2000bp and 2800bp fragments.  Join the 150/SalI/ EcoR V /2000 with 172/SalI/SmaI/2800 to produce p178GL3 (G32G).  **GCTCGGAGGACAGTACTCCG**GATATCTCTCTAGACCCATCCTCTAGAGGGGC**CTCGGAGGACAGTACTCCG**GATC |
| 179 | JN244049 | Sal I/ SmaI double digestion of p172GL3 to produce 2000bp and 2800bp fragments.  Sal I/EcoR V double digestion of p151GL3 to produce 2000bp and 2800bp fragments.  Join the 151/SalI/ EcoR V /2000 with 172/SalI/SmaI/2800 to produce p179GL3 (G34G).  **GCTCGGAGGACAGTACTCCG**GATATCTCTCTAGACCCATCTCCTCTAGAGGGGC**CTCGGAGGACAGTACTCCG**GATCCT |
| 180 | JN244050 | Sal I/ SmaI double digestion of p173GL3 to produce 2000bp and 2800bp fragments.  Sal I/EcoR V double digestion of p172GL3 to produce 2000bp and 2800bp fragments.  Join the 172/SalI/ EcoR V /2000 with 173/SalI/SmaI/2800 to produce p180GL3 (G36G).  **GCTCGGAGGACAGTACTCCG**GATATCTACTCTAGACCCATCTCTCTAGACCCGGGC**CTCGGAGGACAGTACTCCG**GATCCT |
| 181 | JN244051 | Sal I/ SmaI double digestion of p172GL3 to produce 2000bp and 2800bp fragments.  Sal I/EcoR V double digestion of p174GL3 to produce 2000bp and 2800bp fragments.  Join the 174/SalI/ EcoR V /2000 with 172/SalI/SmaI/2800 to produce p181GL3 (G38G).  **GCTCGGAGGACAGTACTCCG**GATATCTCTCTAGACCCATCTCTCTAGACCCATCGGGC**CTCGGAGGACAGTACTCCG**GATCCTA |
| 182 | JN244052 | Sal I/ SmaI double digestion of p172GL3 to produce 2000bp and 2800bp fragments.  Sal I/EcoR V double digestion of p175GL3 to produce 2000bp and 2800bp fragments.  Join the 175/SalI/ EcoR V /2000 with 172/SalI/SmaI/2800 to produce p182GL3 (G40G).  **GCTCGGAGGACAGTACTCCG**GATATCTCTCTAGACCCATCTCTCTAGACCCATCAGGGGC**CTCGGAGGACAGTACTCCG**GATCCT |
| 183 | JN244053 | Sal I/ SmaI double digestion of p172GL3 to produce 2000bp and 2800bp fragments.  Sal I/EcoR V double digestion of p176GL3 to produce 2000bp and 2800bp fragments.  Join the 176/SalI/ EcoR V /2000 with 172/SalI/SmaI/2800 to produce p183GL3 (G42G).  **GCTCGGAGGACAGTACTCCG**GATATCTCTCTAGACCCATCTCTCTAGACCCATCAGAGGGGC**CTCGGAGGACAGTACTCCG**GATC |
| 184 | JN244054 | Sal I/ SmaI double digestion of p172GL3 to produce 2000bp and 2800bp fragments.  Sal I/EcoR V double digestion of p177GL3 to produce 2000bp and 2800bp fragments.  Join the 177/SalI/ EcoR V /2000 with 172/SalI/SmaI/2800 to produce p184GL3 (G44G).  **GCTCGGAGGACAGTACTCCG**GATATCTCTCTAGACCCATCTCTCTAGACCCATCCTAGAGGGGC**CTCGGAGGACAGTACTCCG**GATC |
| 185 | JN244055 | Sal I/ SmaI double digestion of p172GL3 to produce 2000bp and 2800bp fragments.  Sal I/EcoR V double digestion of p178GL3 to produce 2000bp and 2800bp fragments.  Join the 178/SalI/ EcoR V /2000 with 172/SalI/SmaI/2800 to produce p185GL3 (G46G).  **GCTCGGAGGACAGTACTCCG**GATATCTCTCTAGACCCATCTCTCTAGACCCATCCTCTAGAGGGGC**CTCGGAGGACAGTACTCCG**GATC |
| 186 | JN244056 | Sal I/ SmaI double digestion of p172GL3 to produce 2000bp and 2800bp fragments.  Sal I/EcoR V double digestion of p179GL3 to produce 2000bp and 2800bp fragments.  Join the 179/SalI/ EcoR V /2000 with 172/SalI/SmaI/2800 to produce p186GL3 (G50G).  **GCTCGGAGGACAGTACTCCG**GATATCTCTCTAGACCCATCTCTCTAGACCCATCTCCTCTAGAGGGGC**CTCGGAGGACAGTACTCCG**GATCCT |
| 187 | JN244068 | p187GL3 insert the below to Pst I/EcoR V of p126GL3  187F **GCTCGGAGGACAGTACTCCG**GG**CTCGGAGGACAGTACTCCG**GG**CTCGGAGGACAGTACTCCG**G  187R CCGGAGTACTGTCCTCCGAGCCCGGAGTACTGTCCTCCGAGCCCGGAGTACTGTCCTCCGAGCTGCA  DNA sequencing result show that template187 contains 4 GAL4 sites as below.  TAGAATACAAGCTTGCATGCCTGCAG**CTCGGAGGACAGTACTCCG**GG**CTCGGAGGACAGTACTCCG**GG**CTCGGAGGACAGTACTCCG**GG**CTCGGAGGACAGTACTCCG**GATCCTAGAGGATCCCCAGTCCTATATATACTCG |
| 189 | JN244069 | Annealed and inserted between Pst I/Sma I of p145GL3  189F **GCTCGGAGGACAGTACTCCG**GG**CTCGGAGGACAGTACTCCG**GTT  189R AACCGGAGTACTGTCCTCCGAGCCCGGAGTACTGTCCTCCGAGCTGCA  DNA sequencing result show that template 189 contains 4 GAL4 sites as below.  TATAGAATACAAGCTTGCATGCCTGCAG**CTCGGAGGACAGTACTCCG**GG**CTCGGAGGACAGTACTCCG**GG**CTCGGAGGACAGTACTCCG**GTTGGGC**CTCGGAGGACAGTACTCCG**GATCCTAGAGGATCCCCAGTCCTATATATACTCGCTCTG |
| 190 | JN244070 | Annealed and inserted between Pst I/EcoR V of p126GL3  187F **GCTCGGAGGACAGTACTCCG**GG**CTCGGAGGACAGTACTCCG**GG**CTCGGAGGACAGTACTCCG**G  187R CCGGAGTACTGTCCTCCGAGCCCGGAGTACTGTCCTCCGAGCCCGGAGTACTGTCCTCCGAGCTGCA |
| 191 | JN244071 | Annealed and inserted between Pst I/Sma I of p145GL3  144F **GCTCGGAGGACAGTACTCCG**CCCGGG**CTCGGAGGACAGTACTCCG**G  144R CCGGAGTACTGTCCTCCGAGCCCGGGCGGAGTACTGTCCTCCGAGCTGCA |
| 192 | JN244072 | Annealed and inserted between Pst I/Sma I of p145GL3  189F **GCTCGGAGGACAGTACTCCG**GG**CTCGGAGGACAGTACTCCG**GTT  189R AACCGGAGTACTGTCCTCCGAGCCCGGAGTACTGTCCTCCGAGCTGCA |
| 193 | JN244057 | Annealed and inserted between Pst I/EcoR V of p128GL3  142F **GCTCGGAGGACAGTACTCCG**GG**CTCGGAGGACAGTACTCCG**G  142R CCGGAGTACTGTCCTCCGAGCCCGGAGTACTGTCCTCCGAGCTGCA |
| 194 | JN244058 | Annealed and inserted between Pst I/EcoR V of p128GL3  143F **GCTCGGAGGACAGTACTCCG**CCGG**CTCGGAGGACAGTACTCCG**G  143R CCGGAGTACTGTCCTCCGAGCCGGCGGAGTACTGTCCTCCGAGCTGCA |
| 195 | JN244059 | Annealed and inserted between Pst I/EcoR V of p128GL3  144F **GCTCGGAGGACAGTACTCCG**CCCGGG**CTCGGAGGACAGTACTCCG**G  144R CCGGAGTACTGTCCTCCGAGCCCGGGCGGAGTACTGTCCTCCGAGCTGCA |
| 196 | JN244060 | Annealed and inserted between Pst I/EcoR V of p128GL3  145F **GCTCGGAGGACAGTACTCCG**GCCCGGGC**CTCGGAGGACAGTACTCCG**G  145R CCGGAGTACTGTCCTCCGAGGCCCGGGCCGGAGTACTGTCCTCCGAGCTGCA |
| 197 | JN244061 | Annealed and inserted between Pst I/Sma I of p196GL3  123F **GCTCGGAGGACAGTACTCCG**GATATC  146R GATATCCGGAGTACTGTCCTCCGAGCTGCA |
| 198 | JN244062 | Annealed and inserted between Pst I/Sma I of p196GL3  124F **GCTCGGAGGACAGTACTCCG**GATATCAG  147R CTGATATCCGGAGTACTGTCCTCCGAGCTGCA |
| 199 | JN244063 | Annealed and inserted between Pst I/Sma I of p196GL3  125F **GCTCGGAGGACAGTACTCCG**GATATCAGAG  148R CTCTGATATCCGGAGTACTGTCCTCCGAGCTGCA |
| 200 | JN244064 | Annealed and inserted between Pst I/Sma I of p196GL3  126F **GCTCGGAGGACAGTACTCCG**GATATCCTAGAG  149R CTCTAGGATATCCGGAGTACTGTCCTCCGAGCTGCA |
| 201 | JN244065 | Annealed and inserted between Pst I/Sma I of p196GL3  127F **GCTCGGAGGACAGTACTCCG**GATATCCTCTAGAG  150R CTCTAGAGGATATCCGGAGTACTGTCCTCCGAGCTGCA |
| 202 | JN244066 | Annealed and inserted between Pst I/Sma I of p196GL3  128F **GCTCGGAGGACAGTACTCCG**GATATCTCCTCTAGAG  151R CTCTAGAGGAGATATCCGGAGTACTGTCCTCCGAGCTGCA |
| 203 | JN244067 | Annealed and inserted between Pst I/Sma I of p196GL3  173F **GCTCGGAGGACAGTACTCCG**GATATCTACTCTAGACCC  173R GGGTCTAGAGTAGATATCCGGAGTACTGTCCTCCGAGCTGCA |
