# Supplementary material for: Distance and Helical Phase Dependence of Synergistic Transcription Activation in cis-Regulatory Module
Source: PLoS One. 2012 Jan 27;7(1):e31198. doi: 10.1371/journal.pone.0031198 (PMC3267773; doi:10.1371/journal.pone.0031198)
Supplement: Table S2 — Information of DNA molecules used for computational structure construction. (DOC) [file pone.0031198.s003.doc]

Table S2

| NO. | spacings | Accession No. | DNA sequence |
| --- | --- | --- | --- |
| G2 | G0G |  | **CCTCGGAGGACAGTACTCCGCTCGGAGGACAGTACTCCG**A |
| 142 | G2G |  | **GCTCGGAGGACAGTACTCCG**GG**CTCGGAGGACAGTACTCCG**G |
| 143 | G4G |  | **GCTCGGAGGACAGTACTCCG**CCGG**CTCGGAGGACAGTACTCCG**G |
| 144 | G6G |  | **GCTCGGAGGACAGTACTCCG**CCCGGG**CTCGGAGGACAGTACTCCG**G |
| 145 | G8G |  | **GCTCGGAGGACAGTACTCCG**GCCCGGGC**CTCGGAGGACAGTACTCCG**G |
| 146 | G10G |  | **GCTCGGAGGACAGTACTCCG**GATATCGGGC**CTCGGAGGACAGTACTCCG**G |
| 147 | G12G |  | **GCTCGGAGGACAGTACTCCG**GATATCAGGGGC**CTCGGAGGACAGTACTCCG**G |
| 148 | G14G |  | **GCTCGGAGGACAGTACTCCG**GATATCAGAGGGGC**CTCGGAGGACAGTACTCCG**G |
| 149 | G16G |  | **GCTCGGAGGACAGTACTCCG**GATATCCTAGAGGGGC**CTCGGAGGACAGTACTCCG**G |
| 150 | G18G |  | **GCTCGGAGGACAGTACTCCG**GATATCCTCTAGAGGGGC**CTCGGAGGACAGTACTCCG**G |
| 151 | G20G |  | **GCTCGGAGGACAGTACTCCG**GATATCTCCTCTAGAGGGGC**CTCGGAGGACAGTACTCCG**G |
| 172 | G21G |  | **GCTCGGAGGACAGTACTCCG**GATATCTCTCTAGACCCGGGC**CTCGGAGGACAGTACTCCG**G |
| 173 | G22G |  | **GCTCGGAGGACAGTACTCCG**GATATCTACTCTAGACCCGGGC**CTCGGAGGACAGTACTCCG**G |
| 174 | G24G |  | **GCTCGGAGGACAGTACTCCG**GATATCTCTCTAGACCCATCGGGC**CTCGGAGGACAGTACTCCG**G |
| 175 | G26G |  | **GCTCGGAGGACAGTACTCCG**GATATCTCTCTAGACCCATCAGGGGC**CTCGGAGGACAGTACTCCG**G |
| 176 | G28G |  | **GCTCGGAGGACAGTACTCCG**GATATCTCTCTAGACCCATCAGAGGGGC**CTCGGAGGACAGTACTCCG**G |
| 177 | G30G |  | **GCTCGGAGGACAGTACTCCG**GATATCTCTCTAGACCCATCCTAGAGGGGC**CTCGGAGGACAGTACTCCG**G |
| 178 | G32G |  | **GCTCGGAGGACAGTACTCCG**GATATCTCTCTAGACCCATCCTCTAGAGGGGC**CTCGGAGGACAGTACTCCG**G |
| 179 | G34G |  | **GCTCGGAGGACAGTACTCCG**GATATCTCTCTAGACCCATCTCCTCTAGAGGGGC**CTCGGAGGACAGTACTCCG**G |
| 180 | G36G |  | **GCTCGGAGGACAGTACTCCG**GATATCTACTCTAGACCCATCTCTCTAGACCCGGGC**CTCGGAGGACAGTACTCCG**G |
| 181 | G38G |  | **GCTCGGAGGACAGTACTCCG**GATATCTCTCTAGACCCATCTCTCTAGACCCATCGGGC**CTCGGAGGACAGTACTCCG**G |
| 182 | G40G |  | **GCTCGGAGGACAGTACTCCG**GATATCTCTCTAGACCCATCTCTCTAGACCCATCAGGGGC**CTCGGAGGACAGTACTCCG**G |
| 183 | G42G |  | **GCTCGGAGGACAGTACTCCG**GATATCTCTCTAGACCCATCTCTCTAGACCCATCAGAGGGGC**CTCGGAGGACAGTACTCCG**G |
| 184 | G44G |  | **GCTCGGAGGACAGTACTCCG**GATATCTCTCTAGACCCATCTCTCTAGACCCATCCTAGAGGGGC**CTCGGAGGACAGTACTCCG**G |
| 185 | G46G |  | **GCTCGGAGGACAGTACTCCG**GATATCTCTCTAGACCCATCTCTCTAGACCCATCCTCTAGAGGGGC**CTCGGAGGACAGTACTCCG**G |
| 186 | G48G |  | **GCTCGGAGGACAGTACTCCG**GATATCTCTCTAGACCCATCTCTCTAGACCCATCTCCTCTAGAGGGGC**CTCGGAGGACAGTACTCCG**G |
| Z2 | Z0Z |  | **CGCCACATTAGCAATGCCACATTAGCAATGCC**G |
| 162 | Z2Z |  | **G****GCCACATTAGCAAT**GCCGG**GCCACATTAGCAAT**GCCG |
| 163 | Z4Z |  | **GGCCACATTAGCAAT**GCCCCGG**GCCACATTAGCAAT**GCCG |
| 164 | Z6Z |  | **GGCCACATTAGCAAT**GCCCCCGGG**GCCACATTAGCAAT**GCCG |
| 165 | Z8Z |  | **GGCCACATTAGCAAT**GCCGCCCGGGC**GCCACATTAGCAAT**GCCG |
| 166 | Z10Z |  | **GGCCACATTAGCAAT**GCCGATATCGGGC**GCCACATTAGCAAT**GCCG |
| 167 | Z12Z |  | **GGCCACATTAGCAAT**GCCGATATCAGGGGC**GCCACATTAGCAAT**GCCG |
| 168 | Z14Z |  | **GGCCACATTAGCAAT**GCCGATATCAGAGGGGC**GCCACATTAGCAAT**GCCG |
| 169 | Z16Z |  | **GGCCACATTAGCAAT**GCCGATATCCTAGAGGGGC**GCCACATTAGCAAT**GCCG |
| 170 | Z18Z |  | **GGCCACATTAGCAAT**GCCGATATCCTCTAGAGGGGC**GCCACATTAGCAAT**GCCG |
| 171 | Z20Z |  | **GGCCACATTAGCAAT**GCCGATATCTCCTCTAGAGGGGC**GCCACATTAGCAAT**GCCG |
| 142TATA | G2G |  | **GCTCGGAGGACAGTACTCCG**GG**CTCGGAGGACAGTACTCCG**GATCCTAGAGGATCCCCAGTCCTATATATA |
| 143TATA | G4G |  | **GCTCGGAGGACAGTACTCCG**CCGG**CTCGGAGGACAGTACTCCG**GATCCTAGAGGATCCCCAGTCCTATATATA |
| 144TATA | G6G |  | **GCTCGGAGGACAGTACTCCG**CCCGGG**CTCGGAGGACAGTACTCCG**GATCCTAGAGGATCCCCAGTCCTATATATA |
| 145TATA | G8G |  | **GCTCGGAGGACAGTACTCCG**GCCCGGGC**CTCGGAGGACAGTACTCCG**GATCCTAGAGGATCCCCAGTCCTATATATA |
| 146TATA | G10G |  | **GCTCGGAGGACAGTACTCCG**GATATCGGGC**CTCGGAGGACAGTACTCCG**GATCCTAGAGGATCCCCAGTCCTATATATA |
| 147TATA | G12G |  | **GCTCGGAGGACAGTACTCCG**GATATCAGGGGC**CTCGGAGGACAGTACTCCG**GATCCTAGAGGATCCCCAGTCCTATATATA |
| 148TATA | G14G |  | **GCTCGGAGGACAGTACTCCG**GATATCAGAGGGGC**CTCGGAGGACAGTACTCCG**GATCCTAGAGGATCCCCAGTCCTATATATA |
| 149TATA | G16G |  | **GCTCGGAGGACAGTACTCCG**GATATCCTAGAGGGGC**CTCGGAGGACAGTACTCCG**GATCCTAGAGGATCCCCAGTCCTATATATA |
| 150TATA | G18G |  | **GCTCGGAGGACAGTACTCCG**GATATCCTCTAGAGGGGC**CTCGGAGGACAGTACTCCG**GATCCTAGAGGATCCCCAGTCCTATATATA |
| 151TATA | G20G |  | **GCTCGGAGGACAGTACTCCG**GATATCTCCTCTAGAGGGGC**CTCGGAGGACAGTACTCCG**GATCCTAGAGGATCCCCAGTCCTATATATA |
| 173TATA | G22G |  | **GCTCGGAGGACAGTACTCCG**GATATCTACTCTAGACCCGGGC**CTCGGAGGACAGTACTCCG**GATCCTAGAGGATCCCCAGTCCTATATATA |
| 193TATA | G2G |  | G**CTCGGAGGACAGTACTCCG**GG**CTCGGAGGACAGTACTCCG**GATCTCCTCTAGAGGATCCCCAGTCCTATATATA |
| 194TATA | G4G |  | G**CTCGGAGGACAGTACTCCG**CCGG**CTCGGAGGACAGTACTCCG**GATCTCCTCTAGAGGATCCCCAGTCCTATATATA |
| 195TATA | G6G |  | G**CTCGGAGGACAGTACTCCG**CCCGGG**CTCGGAGGACAGTACTCCG**GATCTCCTCTAGAGGATCCCCAGTCCTATATATA |
| 196TATA | G8G |  | G**CTCGGAGGACAGTACTCCG**GCCCGGGC**CTCGGAGGACAGTACTCCG**GATCTCCTCTAGAGGATCCCCAGTCCTATATATA |
| 197TATA | G10G |  | G**CTCGGAGGACAGTACTCCG**GATATCGGGC**CTCGGAGGACAGTACTCCG**GATCTCCTCTAGAGGATCCCCAGTCCTATATATA |
| 198TATA | G12G |  | G**CTCGGAGGACAGTACTCCG**GATATCAGGGGC**CTCGGAGGACAGTACTCCG**GATCTCCTCTAGAGGATCCCCAGTCCTATATATA |
| 199TATA | G14G |  | G**CTCGGAGGACAGTACTCCG**GATATCAGAGGGGC**CTCGGAGGACAGTACTCCG**GATCTCCTCTAGAGGATCCCCAGTCCTATATATA |
| 200TATA | G16G |  | G**CTCGGAGGACAGTACTCCG**GATATCCTAGAGGGGC**CTCGGAGGACAGTACTCCG**GATCTCCTCTAGAGGATCCCCAGTCCTATATATA |
| 201TATA | G18G |  | G**CTCGGAGGACAGTACTCCG**GATATCCTCTAGAGGGGC**CTCGGAGGACAGTACTCCG**GATCTCCTCTAGAGGATCCCCAGTCCTATATATA |
| 202TATA | G20G |  | G**CTCGGAGGACAGTACTCCG**GATATCTCCTCTAGAGGGGC**CTCGGAGGACAGTACTCCG**GATCTCCTCTAGAGGATCCCCAGTCCTATATATA |
| 203TATA | G22G |  | G**CTCGGAGGACAGTACTCCG**GATATCTACTCTAGACCCGGGC**CTCGGAGGACAGTACTCCG**GATCTCCTCTAGAGGATCCCCAGTCCTATATATA |
| 187TATA | G2G2G2G |  | G**CTCGGAGGACAGTACTCCG**GG**CTCGGAGGACAGTACTCCG**GG**CTCGGAGGACAGTACTCCG**GG**CTCGGAGGACAGTACTCCG**GATCCTAGAGGATCCCCAGTCCTATATATA |
| 189TATA | G2G2G7G |  | G**CTCGGAGGACAGTACTCCG**GG**CTCGGAGGACAGTACTCCG**GG**CTCGGAGGACAGTACTCCG**GTTGGGC**CTCGGAGGACAGTACTCCG**GATCCTAGAGGATCCCCAGTCCTATATATA |
| 190TATA | G2G2G |  | G**CTCGGAGGACAGTACTCCG**GG**CTCGGAGGACAGTACTCCG**GG**CTCGGAGGACAGTACTCCG**GATCCTAGAGGATCCCCAGTCCTATATATA |
| 191TATA | G6G5G |  | G**CTCGGAGGACAGTACTCCG**CCCGGG**CTCGGAGGACAGTACTCCG**GGGGC**CTCGGAGGACAGTACTCCG**GATCCTAGAGGATCCCCAGTCCTATATATA |
| 192TATA | G2G7G |  | G**CTCGGAGGACAGTACTCCG**GG**CTCGGAGGACAGTACTCCG**GTTGGGC**CTCGGAGGACAGTACTCCG**GATCCTAGAGGATCCCCAGTCCTATATATA |
